# Thiazolidinediones on PPAR**γ**: The Roles in Bone Remodeling

**DOI:** 10.1155/2011/867180

**Published:** 2011-10-29

**Authors:** Wei Wei, Yihong Wan

**Affiliations:** Department of Pharmacology, UT Southwestern Medical Center, Dallas, TX 75390, USA

## Abstract

Thiazolidinediones (TZDs) are synthetic PPAR**γ** (peroxisome proliferator-activated receptor gamma) agonists and a class of drugs for diabetes mellitus type 2 that can decrease blood sugar efficiently by enhancing insulin sensitivity. However, increased bone fracture risk in diabetic individuals treated with TZDs is one of the reported side effects. Recent studies show that TZDs such as rosiglitazone simultaneously inhibit osteoblast differentiation and activate osteoclast differentiation, leading to bone loss due to decreased bone formation and increased bone resorption. Furthermore, TZDs may activate PPAR**γ** in tissues other than bone, such as the hypothalamus-pituitary-gonad (HPG) axis to indirectly regulate bone mass. This paper will focus on current new developments that implicate potential mechanisms for how PPAR**γ** modulates skeletal homeostasis and how TZDs exert bone-loss side effects.

## 1. Introduction

Rather than a rigid and dormant organ that merely serves an inert support for the vertebrates, bone is a highly dynamic tissue that undergoes constant remodeling, adaptation, repair, and regeneration. Evolution has crafted the structure of the skeletal system in such an elegant way that it is light weighted so birds can fly, yet strong so cheetah can run and hunt. The quantity and quality of bone are influenced by both genetic traits and environmental factors such as nutrition, exercise, and hormone. Bone loss or osteoporosis occurs in physiological and pathological conditions such as aging, postmenopause, sedentary lifestyle, weightlessness during spaceflight, diabetes, and bone metastasis of cancers. With the extension of lifespan and changes in life style, osteoporosis and bone fractures are becoming a leading cause of morbidity and mortality in the modern society. It is estimated that an osteoporotic fracture occurs every 3 seconds worldwide [1]; one out of three women and one out of five men over 50 years of age will experience osteoporotic fractures [2–4]. Between 1990 and 2000, there was nearly a 25% increase in hip fractures worldwide [1]; by 2050, the worldwide incidence of hip fracture is projected to increase by 310% in men and 240% in women [5]. Osteoporosis takes a huge personal and economic toll, for example, in women over 45 years of age, it accounts for more days spent in hospital than many other diseases including diabetes, myocardial infarction, or breast cancer [6]. However, the great majority (>80%) of high-risk individuals are neither identified nor treated [7]. Therefore, enhanced understanding of bone biology and development of effective diagnosis, prevention, and treatment for osteoporosis are of paramount medical urgency and clinical significance.

Bone harnesses an enormous ability to repair and regenerate—both quantity and quality of the bone are maintained by an intricate cellular network composed mainly of three cell types: osteoclasts, osteoblasts, and osteocytes. Osteoclasts are bone-resorbing cells responsible for removing old bones, which are differentiated from hematopoietic progenitors of the monocyte/macrophage lineages upon activation by cytokines such as RANKL (receptor activator of nuclear factor kappa-B ligand) [8–12]. Osteoblasts are bone-forming cells responsible for generating and mineralizing new bone, which are differentiated from mesenchymal progenitors upon activation of several transcription factors such as runx2 and osterix [13–15]. Osteocytes are the most abundant cells in the bone matrix that are thought to derive from osteoblasts, and regulate numerous functions including mechanical sensing, bone remodeling, and mineral metabolism, by both physical interaction and paracrine/endocrine signaling [16, 17]. The balance between bone resorption by osteoclasts and bone formation by osteoblasts is critical for skeletal homeostasis, and bone loss occurs when resorption outpaces formation.

## 2. TZDs Cause Bone Loss and Higher Fracture Rates in Diabetic Patients

Thiazolidinediones (TZDs—troglitazone, rosiglitazone, pioglitazone, and netoglitazone) are widely used for the management of diabetes mellitus type 2. On the one hand, TZDs can enhance glucose uptake, by increasing insulin-sensitivity of adipocytes, muscle, liver, macrophages, and so forth; on the other hand, TZDs can also inhibit gluconeogenesis in the liver, both resulting in decreased insulin resistance and lower blood sugar levels [18, 19]. However, as a common sense, drugs are often accompanied with side effects and TZDs is not an exception. In addition to side effects such as weight gain and fluid retention, increasing reports indicate that both rosiglitazone and pioglitazone are associated with a higher fracture risk. With 4 years of rosiglitazone treatment, type 2 diabetic patients in ADOPT (A Diabetes Outcome Progression Trial) showed increased incidence of fractures in women [20]. Similarly, pioglitazone is also reported to have the same side effect [21]. Moreover, by taking paired stored baseline and 12-month serum samples from 1605 participants (689 women, 916 men) in ADOPT, a recent study showed that CTX-1 (C-terminal telopeptide for type 1 collagen), a marker for osteoclast activity and bone resorption, was increased by 6.1% in the rosiglitazone-treated group in women but not men; P1NP (procollagen type 1 N-propeptide) and bone alkaline phosphatase, two markers for osteoblast activity and bone formation, were decreased in both women and men treated with rosiglitazone [22]. This indicates that elevated bone resorption and suppressed bone formation are two important mechanisms that contribute to the bone loss side effects and the higher fracture risk in women taking TZDs [22]. 

**Figure 1:**
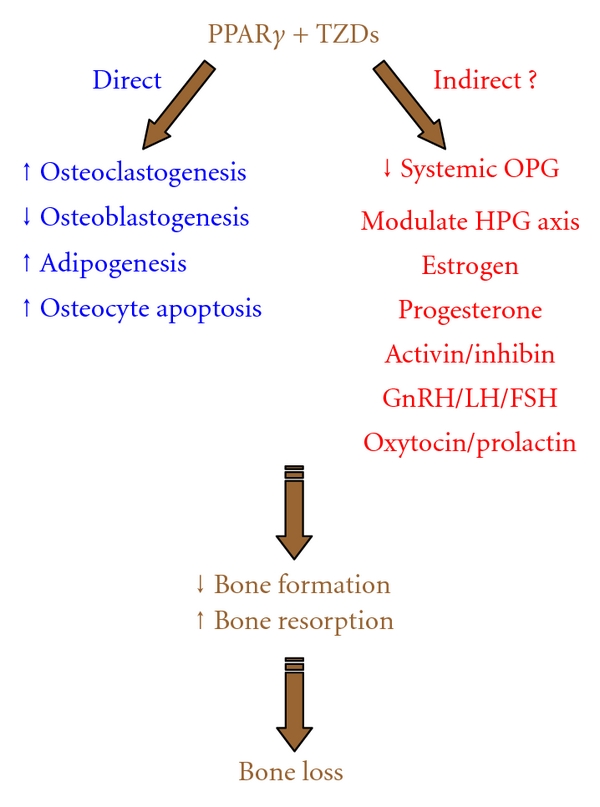
Potential mechanisms for TZD-induced bone loss. In vivo studies in both clinical trials and using animal models demonstrate that TZDs, a class of diabetic drugs that functions as PPAR*γ* agonists, cause bone loss and increased fracture risk, especially in postmenopausal women, by simultaneously inhibiting bone formation and stimulating bone resorption. In addition to the well-documented direct effects of TZDs on bone cell differentiation and function, emerging evidence indicate that TZD may also exert its detrimental skeletal effects via several potential indirect effects, which provokes further investigation in future preclinical and clinical studies.

Importantly, emerging evidence indicates that metabolic state may influence the effects of TZDs on bone [23]. For example, epidemiological studies suggest that skeletal fragility is already increased in type 2 diabetes mellitus [24, 25], potentially due to the inhibition of osteoblast differentiation and function by hyperglycemia-associated ROS (reactive oxygen species) accumulation and/or glucose toxicity [26–28]. Thus, rosiglitazone-induced bone loss may be exacerbated in diabetic patients compared with healthy individuals. An important question for future study is whether and how different metabolic states such as obesity, diabetes, insulin resistance, and aging modulate the relative effects of TZDs on bone resorption versus formation. The purpose of this review is to survey in vivo and in vitro evidence supporting direct and indirect effects of TZDs on osteoclasts and osteoblasts, thus proposing hypotheses for future investigations (Figure 1).

## 3. PPAR*γ* Regulates Osteoblastogenesis and Osteoclastogenesis

PPAR*γ* (peroxisome proliferator-activated receptor gamma) is a member of the nuclear receptor superfamily of transcription factors that can be activated by lipophilic ligands [29]. Upon ligand activation, PPAR*γ* typically induces target gene expression by binding to PPAR response elements (PPREs) in the promoter regions as a heterodimer with the retinoid X receptor (RXR) and recruiting transcription coactivators [29]. Accumulating evidence demonstrates that PPAR*γ* functions as a key regulator of skeletal homeostasis by directly regulating the differentiation of bone cells. 

It has been first reported that PPAR*γ* exerts negative effects on osteoblast differentiation and bone formation. Homozygous PPAR*γ*-deficient embryonic stem cells failed to differentiate into adipocytes, but spontaneously differentiated into osteoblasts; heterozygous PPAR*γ*-deficient mice exhibited high bone mass, resulting from enhanced osteoblastogenesis from bone marrow mesenchymal stem cells but reduced adipogenesis [30]. Consistently, PPAR*γ* activation by TZD treatment inhibits osteoblast differentiation but promotes adipocyte differentiation [31–33]. Moreover, recent evidence has revealed that TZDs may also negatively impact bone by inducing osteocyte apoptosis and the expression of sclerostin, a Wnt antagonist and an inhibitor of bone formation [34, 35].

Subsequently, PPAR*γ* has been found to also enhance RANKL-mediated osteoclast differentiation from hematopoietic stem cells [36]. PPAR*γ* deletion in mouse hematopoietic lineages caused osteoclast deficiency, leading to osteopetrosis manifested as high bone mass and extramedullary hematopoiesis in the spleen, as well as resistance to rosiglitazone-stimulated bone resorption [36]. At molecular levels, PPAR*γ* activation by rosiglitazone suppresses *β*-catenin protein levels and function [37, 38], induces the expression of PGC1*β* (peroxisome proliferator-activated receptor gamma coactivator 1-beta), and potentiates RANKL-induced transcription of c-fos, an essential mediator of osteoclastogenesis [36, 37]. Interestingly, growing evidence suggest that osteoclastogenesis can occur via noncanonical pathways in which RANKL can be substituted by other growth factors such as TNF*α*, LIGHT (TNFSF14), IL-6, TGF*β*, APRIL, BAFF, NGF, IGF-I, or IGF-II [39–46]. In future studies, it would be important to determine the effects of TZD treatment on the osteoclastogenesis mediated by these RANKL-independent pathways.

Pharmacologically, recent clinical trials report that long-term use of TZDs, such as rosiglitazone, increases fracture rates among diabetic patients [20, 22, 24, 47, 48]. Using animal models such as mice and rats, numerous studies show that TZDs cause bone loss by simultaneously decreasing bone formation and increasing bone resorption, thus the uncoupling of bone remodeling [23, 31, 36, 37, 49, 50]. Together, these findings indicate that TZD-induced skeletal fragility is mainly achieved by bone cell-autonomous PPAR*γ* activation, which inhibits osteoblastogenesis and enhances osteoclastogenesis.

## 4. OPG/RANKL in Osteoclastogenesis

The cytokines RANKL (receptor activator of nuclear factor NF-*κ*B ligand) and MCSF (macrophage colony stimulating factor) are required for the differentiation from osteoclasts progenitors into mature osteoclasts. MCSF functions through its receptor MCSFR (c-fms) to promote macrophage/osteoclast precursor proliferation and survival. Upon binding to its receptor RANK, RANKL activates several key transcription factors such as c-fos, c-jun, NFATc1 (nuclear factor of activated T cells, cytoplasmic 1), and NF-*κ*B, as well as osteoclastic enzymes such as TRAP (tartrate-resistant acid phosphatase) and CTSK (cathepsin K), which concertedly trigger the differentiation, multinucleation, maturation, and activation of osteoclasts [12, 51]. As a counter balance, osteoprotegerin (OPG) is a naturally occurring decoy receptor for RANKL, thereby inhibiting osteoclastogenesis and bone resorption [51]. Selectively blocking OPG will increase RANKL activity and osteoclastogenesis, leading to osteoporosis eventually. Both RANKL and OPG are secreted from osteoblasts to regulate osteoclast differentiation; therefore, RANKL/OPG ratio is a critical factor that regulates the balance between osteoblasts and osteoclasts, thus the coupling between bone formation and resorption. 

In addition to PPAR*γ* expressed endogenously in the osteoclasts, PPAR*γ* in other cell types have also been shown to indirectly influence osteoclastogenesis. It has been implicated that PPAR*γ* activation in osteoblast or adipocytes may promote osteoclast differentiation by inducing the expression of RANKL [23, 52]. Another recent study reported that osteoblast-specific overexpression of PPAR*γ* led to lower bone mass in male mice and accelerated ovariectomy-induced bone loss in female mice, associated with not only decreased bone formation but also increased RANKL/OPG ratio [53]. These studies further support the notion that TZDs-induced skeletal fragility is mediated by multifaceted actions of PPAR*γ* on both osteoblast and osteoclast, via both cell-autonomous and paracrine regulations.

Interestingly, recent studies have reported that TZDs can also affect systemic OPG levels. After 24 weeks of treatment of pioglitazone or metformin in 67 type 2 diabetic patients, it was found that plasma levels of OPG decreased in the pioglitazone group, but were unchanged in the metformin group [54]. A similar study showed that comparing 46 type 2 diabetic patients treated with TZDs with 152 type 2 diabetic patients receiving other oral antidiabetes drugs, TZDs treatment is associated with a decrease in plasma OPG levels [55]. Besides in vivo, PPAR*γ* agonists have also been shown to downregulate OPG levels in vitro. Troglitazone can decrease OPG concentration in a dose-dependent manner in 8-day-cultured human mesenchymal stem cells (hMSCs); on the contrary, PPAR*γ* inhibitor GW9662 can enhance early OPG protein levels by hMSCs [56]. Together, these studies have identified RANKL and OPG as potential contributing factors for the enhanced osteoclastogenesis and bone resorption associated with TZDs, suggesting that pharmacological reduction of the RANKL/OPG ratio may be novel strategies to combat TZD-induced bone loss.

## 5. Sex Hormone Receptors and Osteoclast Survival

Estrogen is osteoprotective, and estrogen decline plays an important role in postmenopausal osteoporosis. First, estrogen is implied to decrease the osteoclastic resorption pit in bone by regulating several transcription factors (c-fos, c-jun, e.g.) and inflammatory cytokines (IL-1RI, IL-1RII, e.g.) [57]. Second, our recent study reveals that estrogen loss in mice by ovariectomy stimulates osteoclast progenitors to proliferate and differentiate; suggesting estrogen also inhibits osteoclastogenesis in vivo [58]. Third, estrogen has been shown to promote osteoclast apoptosis [59, 60]. This suggests that estrogen antagonizes osteoclast differentiation and function, and potentially suppresses PPAR*γ*-stimulated osteoclastogenesis. Consistently, estrogen has been shown to be protective against TZD-induced bone loss, for example, TZD-induced bone loss is more significant in postmenopausal women [22, 61, 62]; estrogen significantly reduces TZD-induced adipogenesis [63], osteocyte apoptosis, and sclerostin upregulation [34]. Interestingly, a recent study shows that TZDs (rosiglitazone or pioglitazone) also inhibit estrogen synthesis in human granulosa cells by interfering with androgen binding to aromatase, thus directly affecting estrogen production in human ovarian cells [64]. Together, these findings suggest another potential contributing mechanism for TZD-induced osteoclast activation and bone loss, which may involve the suppression of estrogen function and/or production. In future investigations, it would be important to further examine the in vivo effects of TZDs on estrogen synthesis and activity in animal models and in clinical trials.

Among the two estrogen receptor isoforms (ER*α* and ER*β*), ER*α* is mainly responsible for estrogen regulation of osteoclast survival [60]. ER*α* diminishes mature osteoclast lifespan through the induction of the proapoptotic Fas ligand (FasL) [60]. Selective deletion of ER*α* in differentiated osteoclasts in the ER*α*ΔOc/ΔOc female mice led to a lower bone mass, mimicking the postmenopausal osteoporosis in women [60]. In contrast, in the functional FasL knockout mice (FasLgld/gld), neither enhanced bone resorption nor bone loss was induced by osteoclastic ER*α* deficiency [60]. Thus, in differentiated osteoclast, FasL expression appears to be positively controlled by activated ER*α*. In addition to the role for FasL in estrogen-induced osteoclast apoptosis by an autocrine mechanism involving osteoclasts alone [60], another study has proposed a paracrine mechanism in which estrogen affects osteoclast survival through the upregulation of FasL in osteoblasts (and not osteoclasts) leading to the apoptosis of preosteoclasts [59]. 

A recent study has shown that in two hormone-dependent breast cancer cell lines (MCF-7 and ZR-75-1), PPAR*γ* activation could lead to ER*α* downregulation through the proteasome-dependent degradation pathway [65]; yet different PPAR*γ* agonists exerted differential effects on ER*α* stability: troglitazone, ciglitazone, and natural PPAR*γ* ligand 15d-PGJ(2) induced ER*α* degradation efficiently while rosiglitazone did not alter ER*α* protein levels [65]. Interestingly, another recent study has shown that in MCF7 cells, rosiglitazone could increase the transactivation of FasL promoter through Sp1 site in a dose-related and PPAR*γ*-dependent manner, which could be abrogated by PPAR*γ* antagonist GW9662 [66]. It concludes that PPAR*γ* triggers apoptotic events in breast cancer cells via Fas/FasL signaling pathway [66]. In osteoclasts, the effects of PPAR*γ* activation on ER*α* degradation and FasL expression are underexplored, thus begging the question whether TZDs promote osteoclast survival by reducing ER*α* protein, or promote osteoclast apoptosis by inducing FasL mRNA. Therefore, to determine how PPAR*γ* regulates osteoclast activity, it would be important to investigate both cell-autonomous effects of TZDs on ER*α*/FasL pathway and osteoclast survival; as well as systemic effects of TZDs on estrogen production and its induction of osteoclast apoptosis. 

In addition to ER*α*, there are other transcription factors that potentially regulate the functions of estrogen. For example, the orphan nuclear receptor estrogen-related receptor *α* (ERR*α*) has been shown to regulate many of the same genes as ER*α* [67, 68], and modulate the activity of ER*α* in various tissues including breast, uterus, and bone [69]. ERR*α* has been shown to be involved in the control of not only energy metabolism but also skeletal homeostasis. The roles of ERR*α* in osteoblastogenesis are being actively investigated, and conflicting results have been reported that ERR*α* can either activate or inhibit osteoblast differentiation [70]. Furthermore, our recent study reveals another novel function for ERR*α* in enhancing osteoclastogenesis by inducing the expression of mitochondrial genes via a PGC1*β*-dependent pathway [37]. Induced by PPAR*γ* and coactivated by PGC1*β*, ERR*α* not only promotes mitochondrial activation but also increase osteoclast differentiation [12, 37]. On the one hand, reactive oxygen species (ROS), released from mitochondria, can stimulate osteoclast differentiation by promoting Ca^2+^ oscillations and NFATc1 activation; on the other hand, several transcription factors induced during osteoclastogenesis can activate the expression of target genes required for mitochondrial biogenesis [12, 37, 71]. Consequently, ERR*α* knockout mice exhibit decreased osteoclasts number, bone resorption, and higher bone mass [37]. Because of the crucial role of estrogen deficiency in postmenopausal osteoporosis, it will be interesting in future studies to determine whether and how the PPAR*γ*/PGC1*β*/ERR*α* pathway interacts with the ER signaling in bone remodeling.

## 6. Hypothalamic-Pituitary-Gonadal (HPG) Axis and Bone Remodeling

Although TZDs have been shown to modulate skeletal homeostasis by directly activating PPAR*γ* in bone cells and regulating osteoblast/osteoclast differentiation, new evidence implies that TZDs may also function indirectly through PPAR*γ* in other tissues such as the hypothalamus. A recent study has unveiled an unknown role for central nervous system (CNS) PPAR*γ* in the regulation of energy balance [72]: both acute and chronic activation of CNS PPAR*γ*, by either TZDs or hypothalamic overexpression of a fusion protein consisting of PPAR*γ* and the viral transcriptional activator VP16 (VP16-PPAR*γ*), led to positive energy balance in rats, including cumulative food intake and body fat gain. Blocking the endogenous activation of CNS PPAR*γ* with pharmacological antagonists or reducing its expression with shRNA led to negative energy balance, restored leptin sensitivity in high-fat-diet-fed rats and blocked the hyperphagic response to oral TZD treatment [72]. These findings implicate a provocative hypothesis to be tested in future studies that CNS PPAR*γ* may also contribute to the regulation of bone mass by TZDs, potentially via the hypothalamic-pituitary-gonadal (HPG) relay.

The classic theory predicts that sex steroid (estrogen and androgen) deficiency is the main cause of osteoporosis; however, emerging evidence indicate that other hormones in the hypothalamic-pituitary-gonadal (HPG) axis also participate in the maintenance of bone homeostasis [73]. Progesterone, normally modulated by estrogens, is a female hormone important for the regulation of ovulation, pregnancy, and menstruation. It has been shown that progesterone increases osteoblasts numbers and promotes osteoblasts differentiation and maturation, which is independence of estrogen [74]. Another recent study has reported that the osteoclast differentiation factor RANKL functions as an important in vivo molecular link between progesterone and epithelial carcinogenesis by controlling the incidence and onset of progesterone-driven mammary cancer [75]. This suggests that progesterone may also regulate RANKL-induced osteoclastogenesis. Concerning PPAR*γ*, its agonist, rosiglitazone, can generally antagonize progesterone activity by stimulating progesterone receptor (PR) B degradation and blocking progesterone-induced PRB phosphorylation [76]. Together, these findings suggest that PPAR*γ* regulation of bone homeostasis may be also partially mediated by modulating progesterone function, which remains to be further investigated in future studies.

Activin and inhibin, two closely related protein complexes secreted from the gonad, are members of the transforming growth factor beta (TGF*β*) family, which controls cell proliferation and differentiation in many organs. Activin is either a homodimer composed of two identical *β*A (activin A) or *β*B (activin B) subunits or a heterodimer of *β*A and *β*B (activin AB); inhibin is a heterodimer comprised of *αβ*A (inhibin A) and *αβ*B (inhibin B) subunits, and *α* subunit is unique to inhibin. Activin and inhibin exert opposite biological effects in a variety of cell types. For examples, activin enhances whereas inhibin suppresses follicle-stimulating hormone (FSH) biosynthesis and secretion from the anterior pituitary [77]. Cellular and physiological evidence show that both activins and inhibins regulate osteoblastogenesis and osteoclastogenesis, thus modulating bone mass in vivo [78]. Interestingly, PPAR*γ* has been reported to crosstalk with activin A: on the one hand, activin A treatment can inhibit PPAR*γ* expression in 3T3-L1 preadipocytes, leading to reduced adipocytes differentiation [79]; on the other hand, PPAR*γ* activation by rosiglitazone can decrease follistatin (activin A antagonist) mRNA levels in rat intestinal epithelial cells, leading to the gain-of-function of activin A, which mediates the effects of TZDs on cell proliferation [80]. These results suggest that PPAR*γ* may also interact with the activin/inhibin pathways to regulate bone remodeling, which represents another important future direction for investigation.

Follicle-stimulating hormone (FSH), derived from pituitary, has been proposed to regulate bone mass via several controversial and contradictory mechanisms. In peri-menopausal women, increase of serum FSH, but not loss of estrogen, has been reported to better correlate with bone turnover and/or BMD across the menopause transition [81–83]. It has been shown that FSH enhances osteoclastogenesis by activating MEK/Erk, NF-*κ*B, and Akt; furthermore, FSH*β* KO mice and FSH receptor (FSHR) KO mice are resistant to bone loss despite severe hypogonadism; FSH*β* ^+/−^ mice exhibit increased bone mass and decreased osteoclastic resorption with normal ovarian function, suggesting that the skeletal action of FSH is estrogen independent [84]. In contrast, two recent reports have provided opposite evidence. First, in a prospective clinical study, it has been found that suppression of FSH secretion by a GnRH agonist failed to reduce bone resorption markers in postmenopausal women [85]. Second, using FSH transgenic mice, it has been shown that FSH has dose-dependent anabolic (rather than catabolic) effects on bone, via an ovary-dependent and nonbone-cell-autonomous mechanism [86]. PPAR*γ* is highly expressed in the pituitary [87]. It has been reported that PPAR*γ* and TZDs can alter the secretion and function of pituitary hormones. For examples, pituitary-specific deletion of PPAR*γ* increases luteinizing hormone (LH) levels in female mice and decreases FSH levels in male mice [88]; TZD activation of PPAR*γ* inhibits LH secretion [89] and gonadotropin-releasing hormone (GnRH) signaling [88]. Together, these reports warrant future investigations on the hypothesis that TZDs may partially exert its effects on bone by regulating hormones in the HPG axis such as GnRH, LH, and FSH.

Oxytocin is another pituitary hormone related to the bone homeostasis. Oxytocin has been shown to regulate the differentiation outcome of mesenchymal stem cells by trending toward osteoblasts but inhibiting adipocytes [90]. As oxytocin receptor is expressed and functional in human osteoclasts [91], the possibility exists that oxytocin modulates bone mass by also regulating osteoclastogenesis. Clinically, it is shown that plasma oxytocin levels are significantly lower in postmenopausal women developing osteoporosis than in their healthy counterparts [90]. Mechanistically, oxytocin increases bone morphogenetic protein 2 (BMP2) expression, consequently upregulates the functions of Schnurri-2 and 3, Osterix, and ATF-4, all of which are known activators of osteoblastogenesis [92]. Interestingly, PPAR*γ* has been shown to also crosstalk with oxytocin signaling: in contrast to the aforementioned report [90], oxytocin was reported to stimulate adipocytes differentiation by increasing expression of PPAR*γ*, fatty acid binding protein (FABP), insulin-sensitive glucose transporter 4 (GLUT4), leptin, and CD31 in the epididymal and/or retroperitoneal fat tissue of oxytocin-treated rats [93]. Similar to oxytocin, as another pituitary hormone, prolactin also regulates bone remodeling. In osteoblast-like cells, MG-63, prolactin inhibits osteoblastogenesis and promotes osteoclastogenesis by increasing the ratio of RANKL/OPG [94]. Prolactin signaling has also been shown to interact with PPAR*γ* pathway. On the one hand, prolactin enhances PPAR*γ* and C/EBP*β* mRNA production and augments adipocytes differentiation in NIH-3T3 cells [95]; on the other hand, TZDs activation of PPAR*γ* inhibits prolactin function in pituitary tumor cells [96]. These observations suggest that the effects of TZD and PPAR*γ* on bone remodeling may be also modulated by pituitary hormones such as oxytocin and prolactin, which needs to be further examined in both animal models and clinical investigations in the future.

## 7. Concluding Remarks

In light of the vast array of potential target tissues, pharmacological and mechanistic understanding of cell type-specific gene regulation by PPAR*γ* and TZDs will help to design improved diabetic drugs, such as selective PPAR*γ* modulators, which retain high potency to treat diabetes with minimal bone loss side effects [12, 97]. By using ligands with distinct chemical structures, it has been shown that different PPAR*γ* functions could be separated. Unlike other TZDs, netoglitazone (MCC-555, RWJ-241947) serves as an example of a selective PPAR*γ* modulator that can distinguish the antiosteoblastogenic and proadipogenic functions of PPAR*γ*; mice treated with netoglitazone showed decreased glucose levels similarly to rosiglitazone, but without bone loss [98]. Moreover, a recent study reveals that the proadipogenic effects and insulin-sensitizing effects of PPAR*γ* can be separated using partial agonists that selectively block cdk5-induced PPAR*γ* phosphorylation [99]. As an alternative strategy to improve the outcome of TZD treatment by lessening the side effects, combination therapies haven been used. A recent report suggests that TZD/metformin combination therapy is associated with less weight gain than TZD monotherapy [100]. Thus, if used together with bone anabolic and/or anticatabolic drugs, the TZD-mediated bone loss side effects may be dampened. More provocatively, because PPAR*γ* acts as a double-edged sword to not only inhibit bone formation but also promote bone resorption, bone-specific PPAR*γ* antagonists may represent a potential new therapeutic strategy for the simultaneous anabolic and anticatabolic treatment of osteoporosis.
